# [Corrigendum] Effect of the combination of *Lactobacillus acidophilus* (probiotic) with vitamin K3 and vitamin E on *Escherichia coli* and *Staphylococcus aureus*: An *in vitro* pathogen model and metastasis *in vivo*

**DOI:** 10.3892/mmr.2023.13110

**Published:** 2023-10-05

**Authors:** Ozgur Celebi, Ali Taghizadehghalehjoughi, Demet Celebi, Robin Mesnage, Kirill Sergeevich Golokhvast, Andreea Letitia Arsene, Demetrios A. Spandidos, Aristidis Tsatsakis

Mol Med Rep 27: 119, 2023; DOI: 10.3892/mmr.2023.13006

Subsequently to the publication of the above paper, an interested reader drew to the authors’ attention that, in Fig. 3B on p. 7 showing the results of immunohistochemistry staining experiments, the data panels shown for the ‘L+K’ and ‘EC+E+K’ groups were strikingly similar, such that they appeared to be derived from the same original source, where these panels were intended to show the results from differently performed experiments. The authors have re-examined their original data, and realize that Fig. 3B was inadvertently assembled incorrectly; specifically, the data shown for the ‘L+K’ group in Fig. 3B were featured incorrectly.

The revised version of Fig. 3, now containing the correct data for the ‘L+K’ experimental group in Fig. 3B is shown on the next page. Note that this error did not adversely affect either the results or the overall conclusions reported in this study. All the authors agree with the publication of this corrigendum. They also wish to apologize to the readership of the Journal for any inconvenience caused.

## Figures and Tables

**Figure 3.**
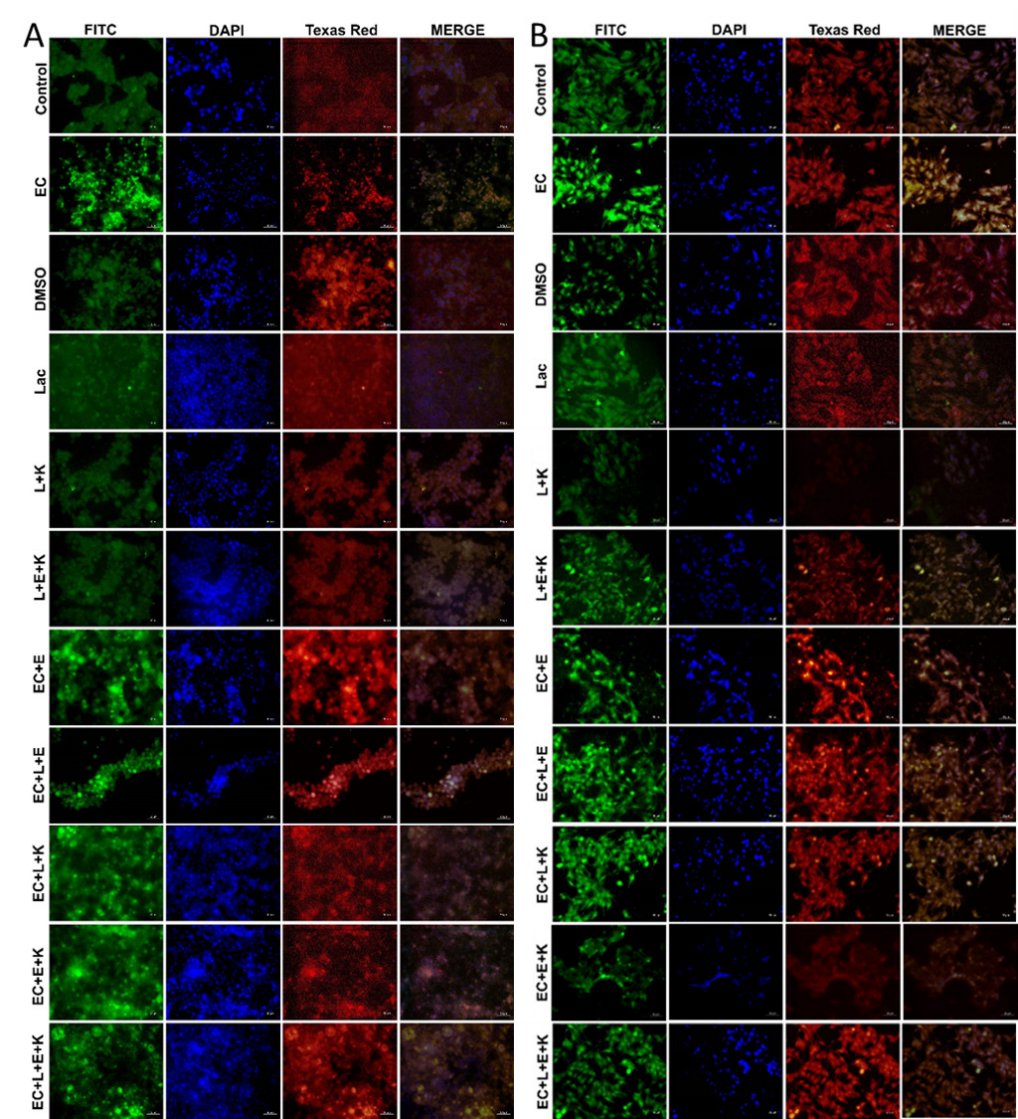
Immunohistochemistry staining. 8-OHdG expression (FITC) and H2A.X expression (Texas Red) in (A) HT-29 and (B) Caco-2 cell lines, immunofluorescence, scale bar, 50 µm. K, vitamin K; E, vitamin E; EC, *Escherichia coli*; L/Lac, *Lactobacillus acidophilus*.

